# Synergistic Effects of Combined Wnt/KRAS Inhibition in Colorectal Cancer Cells

**DOI:** 10.1371/journal.pone.0051449

**Published:** 2012-12-05

**Authors:** Luca Mologni, Stefania Brussolo, Monica Ceccon, Carlo Gambacorti-Passerini

**Affiliations:** 1 Department of Health Sciences, University of Milano-Bicocca, Monza, Italy; 2 Department of Environmental Sciences, University of Ca' Foscari, Venezia, Italy; University of Birmingham, United Kingdom

## Abstract

Activation of Wnt signalling due to inability to degrade β-catenin is found in >85% of colorectal cancers. Approximately half of colon cancers express a constitutively active KRAS protein. A significant fraction of patients show both abnormalities. We previously reported that simultaneous down-regulation of both β-catenin and KRAS was necessary to induce significant cell death and tumor growth inhibition of colorectal cancer cells. Although attractive, an RNAi-based therapeutic approach is still far from being employed in the clinical setting. Therefore, we sought to recapitulate our previous findings by the use of small-molecule inhibitors of β-catenin and KRAS. We show here that the β-catenin inhibitors PKF115-584 and pyrvinium pamoate block β-catenin-dependent transcriptional activity and synergize with the KRAS inhibitor *S-trans, trans*-farnesylthiosalicylic acid (FTS, salirasib) in colon cancer cells driven by Wnt and KRAS oncogenic signals, but not in cells carrying BRAF mutations. The combined use of these compounds was superior to the use of any drug alone in inducing cell growth arrest, cell death, MYC and survivin down-modulation, and inhibition of anchorage-independent growth. Expression analysis of selected cancer-relevant genes revealed down-regulation of CD44 as a common response to the combined treatments. These data provide a proof of principle for a combination therapeutic strategy in colorectal cancer.

## Introduction

Recent advances in our understanding of tumour biology have shown that, despite their great heterogeneity, cancer cells often remain dependent on a limited subset of genetic defects for their survival. The success of targeted therapies in CML, GIST and subgroups of NSCLC clearly indicates that even advanced disease needs the function of its founding oncogenes to grow and survive [1], [2]. This phenomenon, referred to as “oncogene addiction”, offers the basis for targeted cancer therapy, which should ideally be devoid of unwanted side effects on normal cells [3].

Colorectal cancer (CRC) is characterized by well-known genetic defects: the great majority (70–95%) of sporadic CRCs carry mutations that hyper-activate the Wnt pathway, ultimately leading to abnormal β-catenin-dependent gene expression [4], [5], [6], [7]. These alterations occur early during tumour development [8] and likely represent addicting lesions for the tumor. Indeed, down-regulation of β-catenin induces growth arrest and differentiation in CRC cells [9]. However, β-catenin targeting fails to kill the cells [9], [10], [11]. This may be related to the fact that CRCs carry a variety of additional mutations which also appear to be relevant for survival. For instance, KRAS activating mutations are present in approximately 35–50% of colon cancers [4], [6], [12], [13], [14]. If the full complement of Ras pathway members is taken into account, including NRAS, BRAF, NF1, RASSF1A and upstream receptor tyrosine kinases, then 60–80% of the tumours show alteration of the pathway [7], [15], [16]. Genome sequencing data revealed that approximately 30–60% of CRC samples harbour both Wnt and Ras pathway mutations simultaneously [6], [7], [16]. Recent transgenic mouse models of CRC highlighted the importance of both Wnt and KRAS signalling in colon tumourigenesis [15], [17], [18]. Therefore, double targeting might be needed in order to achieve important therapeutic effects.

In our previous work, we demonstrated that combined shRNA-mediated silencing of β-catenin and KRAS in CRC cells led to massive induction of apoptosis *in vitro* and suppression of tumor growth *in vivo*, while individually targeting either of the two pathways showed modest effects [19]. Here, we attempt to translate our findings into a pharmacological approach. Two unrelated compounds with different mechanisms of action, PKF115-584 and pyrvinium pamoate, were used to block β-catenin-dependent transcription. PKF115-584 is a potent and specific small-molecule inhibitor of the β-catenin/Tcf4 interaction and has been validated as an inhibitor of Wnt signalling in different cancer models [20], [21], [22], [23]. Pyrvinium is an anthelmintic drug [24] that has been shown to induce degradation of β-catenin and of its co-factor pygopus, via activation of casein kinase 1α [25]. *S*-*trans*, *trans*-farnesylthiosalicylic acid (FTS, salirasib) has been described as a specific RAS inhibitor [26]. FTS mimics the carboxy-terminal *S*-farnesylcysteine mediating recruitment of RAS proteins to the cell membrane. As a consequence, FTS selectively disrupts the association of chronically active RAS with the membrane, thus blocking its function [27], [28], [29].

We found that combination of the β-catenin inhibitors PKF115-584 and pyrvinium pamoate with the RAS inhibitor FTS synergistically induces growth arrest and apoptosis in CRC cells harbouring both Wnt and KRAS aberrant activation. These data represent a proof of principle for combined Wnt/RAS inhibition in colorectal cancer.

## Materials and Methods

### Cell lines, antibodies and inhibitors

SW837 were a kind gift of Dr. Manuela Gariboldi (IFOM, Milan, Italy) who originally obtained them from ATCC. IFOM Cell Biology Unit confirmed their identity by microsatellite genotyping. All other cell lines were purchased from the American Type Culture Collection, where they are routinely verified using genotypic and phenotypic testing to confirm their identity. Ls174T and HCT-116 cells carry mutations in the CTNNB1 gene that stabilize β-catenin protein [30]. DLD-1, SW480, LoVo and SW837 cells have a truncated APC gene [31], [32]. These six cell lines express a constitutively activated KRAS protein [33], [34], [35]. HT-29 and Colo-201 cell lines have wild-type KRAS but harbour a mutant BRAF^V600E^ allele [33], [36]. Full annotation of these mutations is reported in Table S1. Ls174T cells stably transfected with doxycycline-inducible shRNA constructs were described previously [19]. All cells were maintained in RPMI 1640 medium (except DLD-1 which were grown in DMEM and LoVo in Ham's F12 nutrient) supplemented with 10% foetal bovine serum, 100 units/mL penicillin, 100 μg/mL streptomycin and 2 mmol/L L-glutamine, and incubated at 37°C with 5% CO_2_ atmosphere. Ls174T cells with inducible β-catenin and KRAS shRNA were described previously [19]. The following antibodies were used in this study: anti-KRAS (Abnova, clone 4F3, diluted 1∶1000), anti-pygopus (Santa Cruz biotechnology, H-216, 1∶200), anti-myc (Santa Cruz biotechnology, 9E10, 1∶200), anti-actin (Sigma-Aldrich, 1∶2000), anti-survivin (Santa Cruz biotechnology, D-8, 1∶200), anti-β-catenin (Millipore, 2H4A7, 1∶1000). PKF115-584 was kindly provided by Novartis, Inc. (Basel, Switzerland). *S*-*trans*, *trans*-farnesylthiosalicylic acid (FTS) was synthesized as described [26]. Pyrvinium pamoate was purchased from Sigma-Aldrich. All inhibitors were dissolved in DMSO and stored in small aliquots at −20°C.

### Cell growth and cell death assays

Cell growth and viability was assessed at the indicated times using the CellTiter 96® AQueous One Solution Cell Proliferation Assay System (Promega Corporation) following instructions, as previously reported [37]. To evaluate induction of apoptosis, the cells were seeded in 6-well plates overnight and then treated with inhibitors or vehicle. After 72 hours, the cells were detached by trypsin, washed, and apoptosis was determined using the Annexin V-FITC Apoptosis Detection Kit (Bender MedSystems), according to manufacturer's instructions. All graphs and IC_50_ values were generated using the GraphPad software.

### Dual luciferase assay

The cells in 6-well plate were treated with inhibitors or vehicle and transfected with 2 μg of TOPflash or FOPflash plasmids and 0.1 μg of phRL-CMV (encoding for *Renilla luciferase*, used as an internal control for transfection efficiency) using 3 μl of FuGENE^TM^ 6 reagent. After 24 hours, the cells were harvested, washed, and lysed. Luciferase signals were detected using the Dual-Luciferase® Reporter Assay System (Promega). Firefly luciferase intensity was normalized over Renilla luciferase signal.

### Active KRAS pull-down assay

The cells were treated with FTS or DMSO for 15 hours and then lysed in Magnesium Lysis Buffer (MLB: 25 mM Hepes, pH 7.5, 150 mM NaCl, 10 mM MgCl_2_, 1% NP-40, 0.25% sodium deoxycholate, 10% glycerol) containing protease inhibitors (10 μmol/L benzamidine-HCl and 10 μg/mL each of aprotinin, leupeptin and pepstatin A). Lysates were clarified by centrifugation at maximum speed and quantified by Bradford assay. Equal amount of total proteins (1 mg) were then incubated with 10 μg of Raf-1 RBD agarose beads (Millipore) for 45 minutes at 4°C on a rotating wheel. After 3 washes with MLB, the beads were resuspended in 2X Laemmli sample buffer, boiled and loaded on SDS-PAGE. Active, pulled-down KRAS was revealed by anti-KRAS antibody. Total KRAS (input) was evaluated from the crude lysate.

### Synergism analysis

Cells were treated with vehicle or inhibitors, single or combined at different concentrations and different ratios. After 3 days, the cells were split 1∶10 and reseeded in the presence of inhibitors. On day 6, cell culture growth/viability was monitored by MTS assay. Relative growth, compared to vehicle-treated controls, was used to calculate the fraction affected and the combination index for each experimental combination, using the CalcuSyn software.

### Western blotting

The cells were harvested, washed with ice-cold PBS and resuspended in lysis buffer as described [19]. Total cell extracts were loaded on SDS-PAGE, transferred to a nitrocellulose membrane and probed with the indicated primary antibodies overnight at 4°C. Proteins were revealed by chemiluminescence after incubation with HRP-conjugated secondary antibodies (GE Healthcare, diluted 1∶2500).

### Soft-agar colony assay

Ten thousand cells were embedded in RPMI containing the indicated compounds and 0.3% low-melting agarose type VII (Sigma-Aldrich) and seeded on top of a 0.5% agarose/RPMI support layer in 6-well plates. Fresh inhibitors were added every 7 days to the top agar layer. The colonies were counted after 20 days.

### Quantitative real-time PCR

Cells were treated with vehicle or inhibitors for 24 or 72 hours and harvested. Total RNA was extracted with TRIzol reagent (Invitrogen) and retrotranscribed with random hexamers using a standard procedure. For the 96-well array gene set, forward and reverse primer mix (5 pmol each) was spotted in the corresponding well for each target gene and the plates were kept frozen at −80°C until needed. The reaction mix (2 μl cDNA, 10 μl Brilliant III Ultra-Fast SYBR® Green QPCR Master Mix and 7 μl water, per well) was added to the pre-made plates and quantitative PCR was run on a Stratagene MX3000P detection system, under the following conditions: 95°C, 3 min (1 cycle); 95°C, 10 sec, 60°C, 20 sec (40 cycles). The confirmation run was performed in triplicate using the standard real time PCR protocol recommended by the manufacturer. The GAPDH housekeeping gene was always used as an internal reference. Primers for GAPDH were TGCACCACCAACTGCTTAGC (forward) and GGCATGGACTGTGGTCATGAG (reverse). The primers for all other genes are listed in the Table S2.

## Results

**Figure 1:**
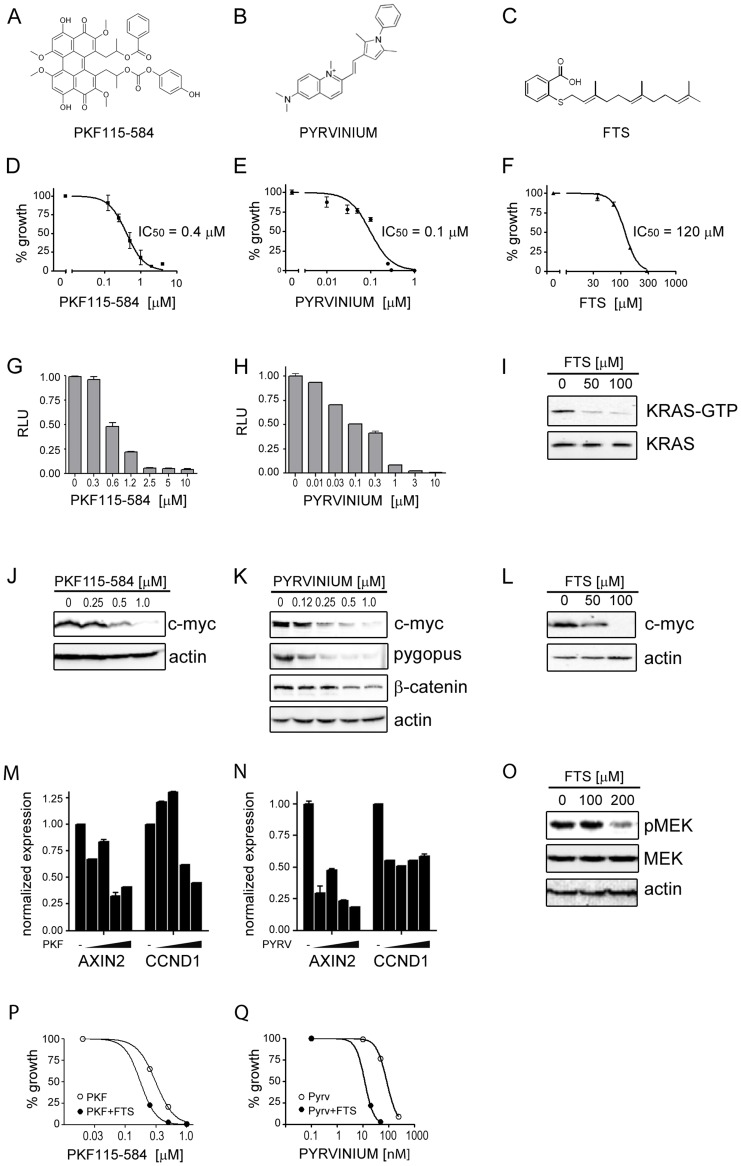
PKF115-584, pyrvinium pamoate and FTS activity in Ls174T cells. (**A–C**) Chemical structures of PKF115-584, pyrvinium and FTS, as previously described (see ref. 20–29) (**D–F**) Dose-response effects of PKF115-584, pyrvinium and FTS on Ls174T cells growth. The cells were exposed at increasing doses of each inhibitor for 72 hours. MTS assay was used to evaluate the effect of the compounds on cell proliferation. IC_50_ values are shown for each compound. (**G–H**) Luciferase activity from the TOPflash plasmid was determined after incubation for 24 hours with PKF115-584 or pyrvinium. Values are Relative Light Units (RLU) with DMSO-treated cells set as 1.00. (**I**) Western blot analysis of active GTP-loaded KRAS pull-down (upper panel) and total KRAS (bottom) from Ls174T cells treated with FTS. (**J–L**) Western blot analysis showing c-myc expression in Ls174T cells treated with increasing concentrations of each compound for 48 hours. From pyrvinium-treated cells, pygopus and β-catenin expression are also shown (**K**). Actin is always shown as a loading control. (**M–N**) Quantitative PCR analysis of AXIN2 and CCND1/cyclin D1 expression after treatment with increasing doses (0.125–1.0 μM) of PKF115-584 (**M**) and pyrvinium (**N**). (**O**) Western blot analysis of MEK phosphorylation in FTS-treated cells. Total MEK and actin are shown as controls. (**P–Q**) Dose-response curves of PKF115-584 and pyrvinium in the absence (empty circles) or presence (filled circles) of 100 μM FTS. Each individual curve is normalized on the corresponding sample with no β-catenin inhibitor.

PKF115-584 (figure 1A) and pyrvinium pamoate (figure 1B) have been shown to interfere with the β-catenin-associated transcriptional complex through different mechanisms. In order to verify the activity of the two drugs in CRC cells, we characterized their effects in the Ls174T cell line, carrying β-catenin and KRAS activating mutations [30], [33]. This cell line was initially chosen as a model because it was previously used to characterize the effects of siRNA-mediated gene silencing [19]. As reported in figure 1D–E, both drugs inhibited cell growth in a dose-dependent manner. Similar growth inhibition was obtained in DLD-1 cells, which express a truncated APC allele (figure S1A–B). Concomitantly, the two compounds inhibited transcription from the β-catenin/Tcf4-responsive reporter plasmid TOPflash (figure 1G–H). The IC_50_ values observed for cell proliferation and TOPflash curves are in agreement, suggesting that growth arrest is mediated by β-catenin inhibition. As expected, pyrvinium induced loss of pygopus expression (figure 1K). The same result was obtained in DLD-1 cells (figure S1E). In addition, pyrvinium has been reported to force β-catenin degradation [25]. Surprisingly, β-catenin expression was unchanged in pyrvinium-treated DLD-1 cells (figure S1E), while it slightly decreased in Ls174T cells (figure 1K). Sequencing analysis of β-catenin gene confirmed the presence of the S45F substitution in Ls174T cells and wild-type sequence in DLD-1 cells within the N-terminal phosphorylation region (figure S2). Both drugs blocked endogenous expression of MYC, a well-known β-catenin transcriptional target and a strong promoter of cell growth (figure 1J–K and figure S1D–E). To confirm inhibition of the Wnt pathway, expression of two additional known target genes was analysed by real-time quantitative PCR. Both AXIN2 and CCND1 (encoding for cyclin D1) genes were down-regulated by treatment with PKF115-584 and pyrvinium (Figure 1M–N).

The RAS inhibitor FTS (figure 1C) inhibited cell growth at high micromolar concentrations (figure 1F and figure S1C), in line with previous reports [38], [39], [40]. FTS depleted the GTP-loaded (active) KRAS pool, while leaving total KRAS amount unchanged (figure 1I). This anti-KRAS activity translated into a marked decrease of MYC protein levels (figure 1L) and MEK phosphorylation (figure 1O), but not of FOS expression (data not shown) in Ls174T cells. However, FTS treatment led to different molecular responses in DLD-1 cells: phospho-MEK signal was unaltered and MYC was only minimally affected, while FOS expression decreased substantially (figure S1F). To assess whether the anti-proliferative activity of β-catenin inhibitors could be potentiated by FTS, dose-response curves were generated by exposing Ls174T cells to increasing doses of PKF115-584 (figure 1P) and pyrvinium pamoate (figure 1Q) in the absence or presence of 100 µM FTS. In both cases, addition of FTS shifted the curve significantly. In particular, sensitivity to pyrvinium increased by about 10-fold (IC_50_ pyrvinium, 0.1 µM; IC_50_ pyrvinium+FTS, 0.01 µM). These results altogether indicate that the anti-proliferative effects of two β-catenin inhibitors and the RAS inhibitor FTS correlate with specific inhibition of β-catenin and KRAS activities, respectively, in Ls174T cells. Furthermore, FTS enhances cytotoxicity of β-catenin inhibitors in these cells.

**Figure 2:**
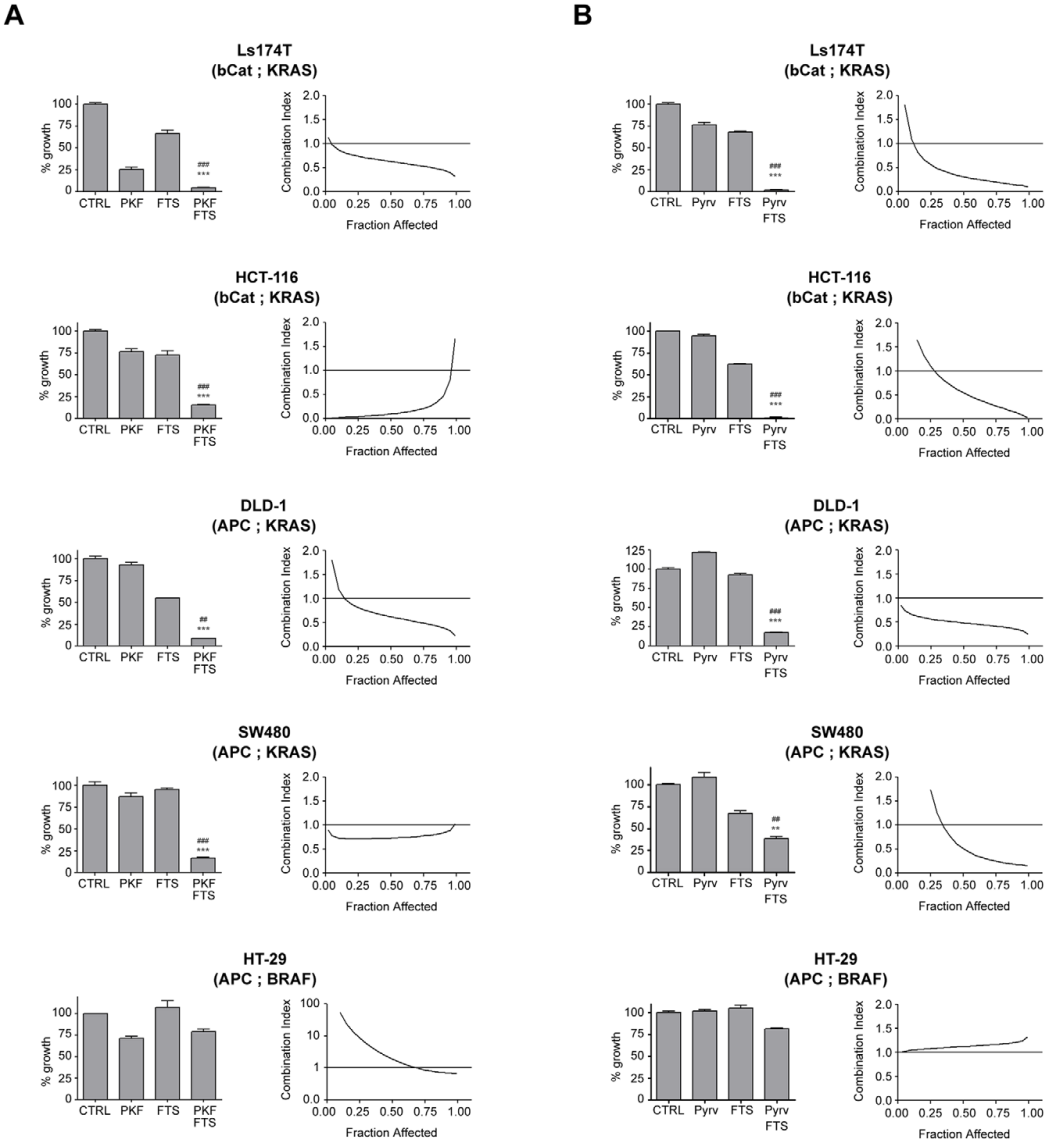
Synergistic effects of PKF115-584/FTS and pyrvinium/FTS combinations on CRC cells. The indicated cell lines were cultured for 6 days in the presence of inhibitors as single agents or in combination, or vehicle alone. MTS assay was used to assess the effects of each treatment on cell culture growth and viability. (**A**) PKF115-584/FTS and (**B**) pyrvinium/FTS combination. For each cell line, the bar graph (**left**) shows the effect at a single dose (PKF115-584, 0.25 μM [Ls174T and DLD-1] or 0.125 μM [SW480 and HCT-116]; pyrvinium, 50 nM; FTS, 100 μM). The XY graph (**right**) shows combination indexes as a function of the fraction affected (see Materials and Methods). For each cell line, the genes that are mutated, affecting Wnt and Ras pathways, are indicated in brackets. CTRL  =  control; Pyrv  =  pyrvinium. In all panels, number symbols (**#**) indicate significance versus the β-catenin inhibitor (PKF115-584 or pyrvinium); asterisks (*) indicates significance versus FTS; one symbol, p<0.05; two symbols, p<0.01; three symbols p<0.001.

In order to better define the cooperation of β-catenin and KRAS inhibition in CRC cells, the activity of PKF115-584 and pyrvinium, alone and in various combinations with FTS, was tested by MTS assay on a panel of CRC cell lines carrying different oncogenic mutations (table S1). Results obtained at fixed concentrations are shown in figure 2 as bar graphs (PKF115-584: 125 or 250 nM; pyrvinium: 50 nM; FTS: 100 µM). Interestingly, while sensitivity to single agents differed among the various cell lines, in all KRAS-mutated cells the combined treatments caused significantly higher growth inhibition compared to single treatments. Synergism was then studied more extensively over a range of inhibitors concentrations, using the method described by Chou and Talalay, based on the mass-action law principle [41]. Combination Index values (CI) were calculated from experimental data along the whole range of fractional effects and are shown as XY plots in figure 2. Specific CI values at ED50, ED75 and ED90 are reported in Table 1. As predicted by oncogene mutation status, all cell lines carrying mutations in both Wnt pathway (APC or β-catenin) and KRAS showed synergistic interactions (CI<1), except at extreme points of fraction affected, where effects are either negligible or too strong. In contrast, HT-29 cells (carrying wild-type KRAS and a mutated BRAF^V600E^ allele) showed no synergism. Rather, antagonistic effects (CI>1) were noted. However, combination of PKF115-584 with a BRAF inhibitor (PLX-4032) showed weak synergism in HT-29 cells at high doses (figure S3). Additional data obtained with pyrvinium/FTS in two KRAS- and one BRAF-mutated cell lines are shown in figure S4. Overall, analysis of CI values at ED50 (pyrvinium/FTS) shows correlation between KRAS/BRAF mutational status and synergistic interaction (p = 0.0021): all KRAS mutant cells showed synergism, while neither of two BRAF mutant cell lines did (figure S5), although the limited number of BRAF mutant cell lines tested does not allow to draw definitive conclusions. In contrast, the effects did not correlate with p53 or MSI status (table 1). Altogether, these results suggest that PKF115-584 and pyrvinium can be combined with FTS in CRC cells harbouring concomitant Wnt/KRAS genetic abnormalities, to achieve better therapeutic effects.

**Table 1:** Combination Index values obtained at ED50, ED75 and ED90. The average of the three values is reported in the last row.

	*PKF115-584 + FTS*					*Pyrvinium + FTS*				
	Ls174T	HCT116	DLD1	SW480	HT29	Ls174T	HCT116	DLD1	SW480	HT29
**ED50**	0.628	0.097	0.621	0.732	1.891	0.312	0.549	0.484	0.508	1.125
**ED75**	0.533	0.214	0.488	0.777	0.870	0.198	0.275	0.415	0.248	1.166
**ED90**	0.452	0.473	0.387	0.839	0.709	0.146	0.138	0.355	0.185	1.211
**Mean**	**0.538**	**0.261**	**0.499**	**0.783**	**1.157**	**0.219**	**0.321**	**0.418**	**0.314**	**1.167**

**Figure 3:**
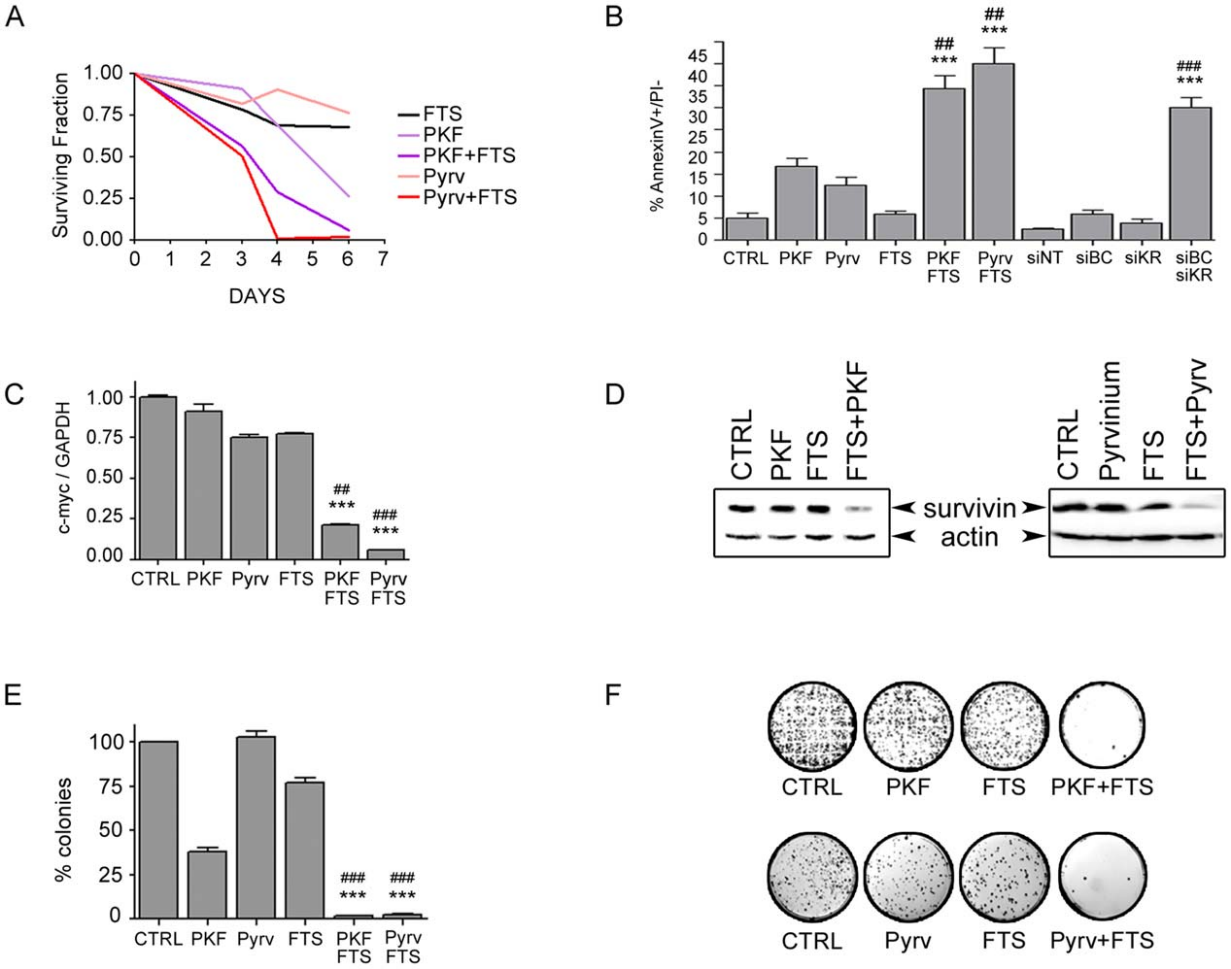
Characterization of synergism in Ls174T cells. (**A**) Cells were treated with 100 μM FTS, 0.25 μM PKF115-584 or 50 nM pyrvinium, alone or in combinations, or vehicle, for 6 days. Cell culture viability was assessed on days 3, 4 and 6 by MTS. The relative OD compared to vehicle-treated control cells was recorded as surviving fraction. (**B**) Ls174T cells were cultured for 3 days in the presence of the indicated compounds (same concentrations as in [**A**]); in parallel, Ls174T cells carrying inducible siRNAs (siNT, non-targeting; siBC, anti-β-catenin; siKR, anti-KRAS) were treated with doxycycline for the same time. Apoptosis was determined by annexin V/propidium iodide double staining. The percentage of early apoptotic cells (annexin V-positive/propidium iodide-negative) is reported in the graph. (**C**) Ls174T cells were treated with PKF115-584 (0.5 μM), pyrvinium (50 nM) and FTS (100 μM), alone or in combination, for 24 hours and c-myc expression was evaluated by real-time PCR using GAPDH as a reference gene. Expression was normalized on vehicle-treated control cells. (**D**) The cells were incubated for 72 hours with inhibitors and cell lysates probed with anti-survivin and β-actin antibodies. (**E–F**) Ten thousand Ls174T cells were grown in soft-agar in the presence of PKF115-584 (0.1 μM), pyrvinium (25 nM), FTS (50 μM), alone or combined. Large colonies were counted 20 days later. The number of colonies formed by untreated control cells is set as 100% (**E**). Representative photographs are shown in panel (**F**). Statistical analysis: see legend to figure 2.

We then further characterized the synergistic combinations using additional independent assays (figure 3 and figure S6). Cell growth was monitored during the course of several days, after exposing Ls174T and DLD-1 cells to the three drugs, alone or in combination, showing that FTS potentiated PKF115-584 and pyrvinium toxicity already after 3–4 days (figure 3A and figure S6A). Growth inhibition was accompanied by induction of apoptosis: the number of early apoptotic cells was significantly higher in the combination samples compared to either drug alone (figure 3B and figure S6B). As a comparison, apoptosis in Ls174T cells was assessed in parallel after induction of anti-β-catenin and anti-KRAS shRNAs, reaching a comparable level of early apoptosis in the double-silenced sample (figure 3B). Silencing of target proteins is shown in figure S7. Next, as all three compounds are able to inhibit MYC expression in Ls174T cells (figure 1J–L), we determined whether combining suboptimal concentrations of the drugs could induce a better blockage of MYC transcription. Previous reports have shown that both KRAS and β-catenin are able to regulate MYC expression in colon cancer cells [19], [42], [43]. Indeed, combination treatments for 24 hours showed a significant improvement in MYC expression inhibition compared to single treatments (figure 3C). This result suggests that MYC is a common effector of the two pathways and its down-regulation correlates with cell growth and viability inhibition in these cells. In contrast, we observed no effect of the combinations on MYC levels in DLD-1 cells (figure S6C), in line with the lack of MYC down-regulation by FTS in these cells. Furthermore, combined inhibition caused strong down-modulation of survivin expression, while no or little change was obtained by single treatments (figure 3D and figure S6D). Survivin is a transcriptional target of both Wnt and KRAS pathways [19], [44], [45] and has a crucial role in the survival of KRAS-driven cancer [46]. Finally, to study the long-term effects of β-catenin and KRAS combined inhibition, anchorage-independent growth was assessed after a single addition of sub-lethal doses of PKF115-584 or pyrvinium, and FTS. As shown in figure 3E–F and in figure S6E–F, combination of each β-catenin inhibitor with FTS had a profound effect on soft-agar growth of Ls174T and DLD-1 cells.

**Figure 4:**
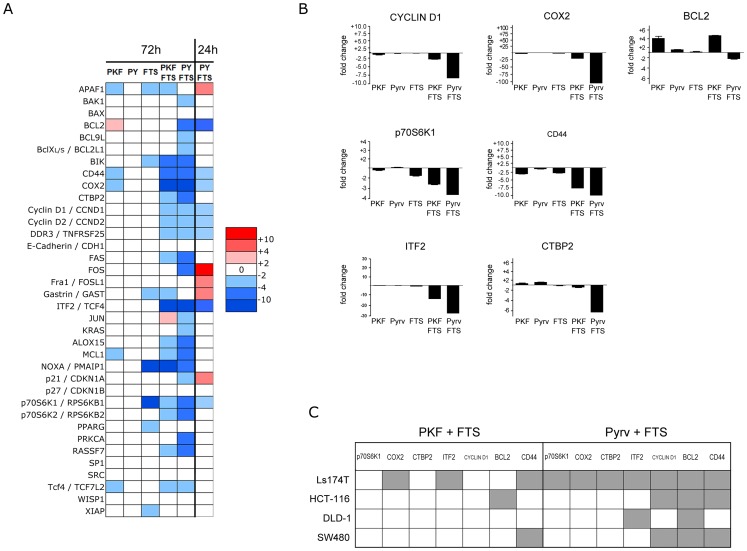
Expression analysis of selected genes after inhibitors treatments. Ls174T cells were treated for 24 or 72 hours with inhibitors and a panel of genes focused on Wnt, Ras and apoptosis pathways was studied by QPCR using a 96-well array (**A**) or standard real-time PCR (**B**). The graphs in (**B**) show expression fold change compared to vehicle-treated controls. (**C**) Summary table reporting real-time PCR data analysis from all cell lines. Filled boxes indicate synergistic effect on gene expression (>2-fold change versus every single treatment). PY  =  pyrvinium.

In order to gain further insight into the transcriptional modifications induced by the combined treatments, the expression of a selected panel of genes related to Wnt and KRAS signalling, colon cancer and apoptosis, was studied using a home-made quantitative PCR array. The heatmap in figure 4A shows relative changes in expression after 72 hours of treatment with single or combined drugs, compared to vehicle-treated control cells. As expected, single agents left most genes unchanged, whereas the combinations induced a general repression of the selected gene set. In particular, CD44, COX2, CTBP2, Cyclins D1 and D2, ITF2, p70S6K2 and RASSF7 were strongly down-regulated by both combinations, compared to single treatments. In addition, the pyrvinium/FTS combination caused down-modulation of additional genes such as BCL2, BCL2L1 (encoding for the Bcl-X_L_ anti-apoptotic factor), BCL9L, KRAS, CDKN1A (p21^WAF1^) and PRKCA. In order to catch early transcriptional changes that occur before any sign of cellular stress, a 24-hour pulse was run with the pyrvinium/FTS combination. This combination was preferred over the one with PKF115-584 for this analysis, as pyrvinium showed a higher degree of gene down-regulation. The data are reported in the right-most column of the heatmap. Despite some differences with the longer drug exposure, many of the changes were conserved, including down-regulation of CD44, COX2, BCL2 and Cyclins D. In addition, up-regulation of the pro-apoptotic protein APAF1 was noted. Interestingly, p21^WAF1^ was transiently up-regulated at 24 hours before being down-regulated at 72 hours. To further confirm these data, a few genes were analysed independently by standard real-time PCR in four cell lines. Figure 4B shows the results from Ls174T cells, confirming gene regulation observed in the original data set. The data from all four cell lines were further analysed to identify synergistic gene expression modulation, defined as >2-fold change of expression in a combination compared to single treatments (figure 4C). Interestingly, BCL2 expression was synergistically down-regulated in all four cell lines by pyrvinium/FTS combination (filled grey boxes). Cyclin D1 and CD44 scored synergistic in three out of four cell lines. CD44 was also down-regulated in DLD-1 cells, but the extent of modulation did not reach the threshold for a synergistic effect (figure S8). The PKF115-584/FTS combination proved less effective in this analysis, with only CD44 showing synergistic down-regulation in at least two cell lines (Ls174T and SW480). These results indicate CD44 as a common response to the double β-catenin/KRAS targeting.

Having proved the efficacy of double Wnt/KRAS inhibition *in vitro*, we tried to translate these findings into a novel therapeutic strategy *in vivo*. However, PKF115-584 is no longer produced by Novartis and its synthesis in large scale proved extremely challenging and time-consuming. As for pyrvinium pamoate, it is reported to be poorly absorbed [47]. However, oral administration of pyrvinium was described by two groups [48], [49]. Therefore, a pilot experiment was run to verify if the drug could reach the target in nude mice, by looking at pygopus expression in Ls174T xenografts after three daily oral administrations of 10 mg/kg pyrvinium. The results were negative (figure S9), therefore we did not attempt further *in vivo* analyses.

## Discussion

Almost all colorectal cancers show alteration of the Wnt pathway that controls β-catenin activation. Therefore, inhibition of β-catenin may lead to significant progress in the management of this disease. However, recent data revealed that more than one “driver” oncogenic pathway is often activated in a single tumor [50], [51]. Therefore, multiple oncogene targeting is likely to be needed to eradicate the disease. In a significant fraction of colorectal tumors, Wnt and KRAS pathway alterations coexist [6], [13], [14]. Despite the fact that it has been known for nearly 30 years, the KRAS oncogene has been an elusive target so far. Agents that block KRAS post-translational modifications have been ineffective in clinical trials [52]. Strategies to target downstream effectors of KRAS [53], [54], [55] or to establish synthetic lethal interactions [56], [57], [58] have shown different responses from case to case, possibly due to the complexity of the KRAS pathway. This is also highlighted by the different molecular outcome of KRAS inhibition observed in two CRC cell lines in our study. Taking a different approach, FTS specifically interferes with KRAS docking to the cell membrane, by mimicking its farnesylcysteine moiety [29].

In this work, the effects of combined β-catenin and KRAS inhibition by small molecules was investigated. Synergism was evident in a panel of CRC cell lines carrying different types of mutations affecting the target pathways, by using several independent assays. Combining sub-optimal doses of β-catenin and KRAS inhibitors we observed significant growth inhibition and apoptosis, which were mirrored by strong down-regulation of survivin expression. Survivin inhibits activation of effector caspases and is up-regulated in many cancers. By hitting two pathways that control its expression in CRC cells, we obtained complete suppression of its anti-apoptotic activity and hence induction of apoptosis. Recently, a molecular classification of CRC cell lines was proposed, based on their dependency on KRAS [57]. However, these and our previous data suggest that so-called KRAS-independent cells rather show double dependency, as they rely on both β-catenin and KRAS to survive. Interestingly, FTS induced differential molecular effects on two Ras-triggered signalling pathways in Ls174T and DLD-1 cells, although the final biological effect was similar. Ls174T cells showed inhibition of MAPK signalling, while DLD-1 cells blocked FOS expression, which is downstream to RalA [59]. This result emphasizes the importance of directly targeting oncogenic RAS proteins rather than their broad and diverse signalling tree. Partial characterization of the transcriptional changes induced by the combined treatments revealed that CD44 was commonly down-regulated by the combinations. CD44 is a transmembrane adhesion molecule acting as the receptor for hyaluronan, a major component of the tumor extracellular matrix. CD44 transcript variants have been shown to be overexpressed and to mediate cell survival in colon cancer. Hence, CD44 has been proposed as a target for CRC therapy [60], [61]. Other genes (RPS6KB1, COX2, CTBP2) showed variable regulation among the different cell lines, suggesting cell line-specific responses. Furthermore, despite many similarities, the two combinations showed some differences in gene regulation. In general, pyrvinium/FTS caused a more pronounced down-regulation of some genes compared to PKF115-584/FTS. This may be due to a more effective β-catenin block, or to off-target effects of pyrvinium. In addition, the PKF115-584/FTS combination showed little synergistic gene modulation, likely because the extent of down-regulation was often less robust compared with pyrvinium/FTS. Interestingly, CDKN1A gene (encoding for the cell-cycle inhibitor p21^WAF1^) showed transient up-regulation followed by repression; p21^WAF1^ protein is known to have a dual role in the response to stress stimuli: it mediates early growth arrest but also protects from apoptosis after prolonged stress [62], [63]. Therefore, its down-modulation at later time points may be needed in order to trigger cell death.

Interestingly, our data on β-catenin modulation are in contrast to the original report [25] showing that pyrvinium pamoate induced its degradation in APC-mutant cells (SW480) but not in β-catenin-mutant cells (HCT-116 WTKO). We found that β-catenin was slightly down-regulated in Ls174T cells, which express a non-phosphorylatable mutant form of β-catenin, but not in DLD-1 cells, expressing wild-type β-catenin. Thus, the effects on β-catenin expression do not seem to correlate with its genetics. More studies are needed to address this point. Clearly, however, the growth inhibitory activity of pyrvimium correlates with down-regulation of pygopus.

In conclusion, this study confirms and extends our previous data showing that concomitant inhibition of two commonly mutated pathways (Wnt and KRAS) leads to superior antitumor effects in colon cancer cells that carry those mutations. The synergistic activity was specific, as it was not observed in CRC cells expressing wild-type KRAS. The potential advantages offered by a synergistic combined treatment resides in the possibility to (i) shut down both oncogenic drivers and (ii) reduce drug doses, thus limiting toxic effects. The obvious limitation of this work is the lack of *in vivo* validation. Unfortunately, the effects of these drug combinations could not be evaluated in mice for technical reasons. Despite this, in our opinion, the *in vitro* results presented here represent a convincing proof-of-principle for double pharmacologic targeting and encourage the development of clinical β-catenin and KRAS inhibitors for targeted cancer therapy.

## Supporting Information

Figure S1 **Effects of PKF115-584, Pyrvinium and FTS treatments in DLD-1 cells.** The cells were treated with increasing concentrations of PKF115-584 (**A**, **D**), pyrvinium (**B**, **E**) or FTS (**C**, **F**). Cell growth was measured at 72 hours by MTS assay (**A–C**). Total lysates were probed with the indicated antibodies (**D–F**).
(TIF)

Figure S2 **Sequence of the N-terminal b-catenin region in DLD-1 and Ls174T cells.** The known CK1α and GSK3β target aminoacids 33, 37, 41 and 45 are indicated. Mutation of Ser45 to Phe is confirmed in Ls174T cells.
(TIF)

Figure S3 **Effects of PKF115-584/PLX-4032 combination in HT-29 cells.** (**A**) The cells were cultured for 6 days in the presence of inhibitors as single agents or in combination (PKF  = 0.5 μM; PLX  = 2 μM). Percent growth was evaluated by MTS assay. (**B**) Combination Index values were calculated by CalcuSyn.
(TIF)

Figure S4 **Synergistic effects of pyrvinium/FTS combination in LoVo, SW837 and Colo-201 cell lines.** The cells were cultured for 6 days in the presence of inhibitors and then assayed by MTS as described in figure 2. Fractional effect graphs confirm synergism in KRAS-mutated cell lines LoVo and SW837 but not in BRAF-mutated Colo-201.
(TIF)

Figure S5 **Summary of Combination Index values at ED50 for the pyrvinium/FTS combination.** BRAF-mutated cell lines are shown in purple.
(TIF)

Figure S6 **Characterization of synergism in DLD-1 cells.** (**A**) Cells were treated with 100 μM FTS, 125 nM PKF115-584 or 50 nM pyrvinium, alone or in combinations, or vehicle, for 7 days. Cell culture viability relative to control was assessed by MTS. (**B**) Apoptosis was determined by annexin V/propidium iodide double staining after 72 hours treatment as indicated. (**C**) c-myc expression evaluated by real-time PCR after 24 hours incubation with the indicated compounds, using GAPDH as a reference gene. Expression was normalized on vehicle-treated control cells. (**D**) The cells were incubated for 72 hours with inhibitors and cell lysates probed with anti-survivin and β-actin antibodies. (**E–F**) Ten thousand cells were grown in soft-agar in the presence of PKF115-584 (0.25 μM), pyrvinium (25 nM), FTS (100 μM), alone or combined. Large colonies were counted 20 days later. The number of colonies formed by untreated control cells is set as 100% (**E**). Representative photographs are shown in panel **F**.
(TIF)

Figure S7 **Western blot analysis of siRNA target genes.** Ls174T cells stably expressing dox-inducible shRNAs were treated for 3 days with doxycycline. Total cell lysates were probed with anti-β-catenin (BCAT), anti-KRAS or anti-actin antibodies. siNT, non-targeting siRNA; siBC, anti-β-catenin siRNA; siKR, anti-KRAS siRNA.
(TIF)

Figure S8 **CD44, Cyclin D1 and BCL2 expression fold change obtained in DLD-1, SW480 and HCT-116 cells.** The cells were treated for 72 hours with the indicated compounds. Gene expression was analyzed by real-time PCR as in figure 4B. PYRV  =  pyrvinium.
(TIF)

Figure S9 **Repeated oral administration of pyrvinium pamoate (10** **mg/kg q.d. for 3** **days) did not cause pygopus down-modulation in nude mice.** Two control mice and two treated mice are shown. A marker lane running in the middle of the gel has been deleted. Actin is shown as a loading control.
(TIF)

Table S1 **Mutations in the genes encoding APC, β-catenin, KRAS, BRAF and p53, and microsatellite instability status (MSI) of CRC cell lines used in this study.**
(DOC)

Table S2 **Primers used for QPCR analysis. The common protein name is provided in brackets along with the official gene symbol, when different.**
(DOC)
