# Supplementary figures and images for: Synergistic Effects of Combined Wnt/KRAS Inhibition in Colorectal Cancer Cells
Source: PLoS One. 2012 Dec 5;7(12):e51449. doi: 10.1371/journal.pone.0051449 (PMC3515485; doi:10.1371/journal.pone.0051449)

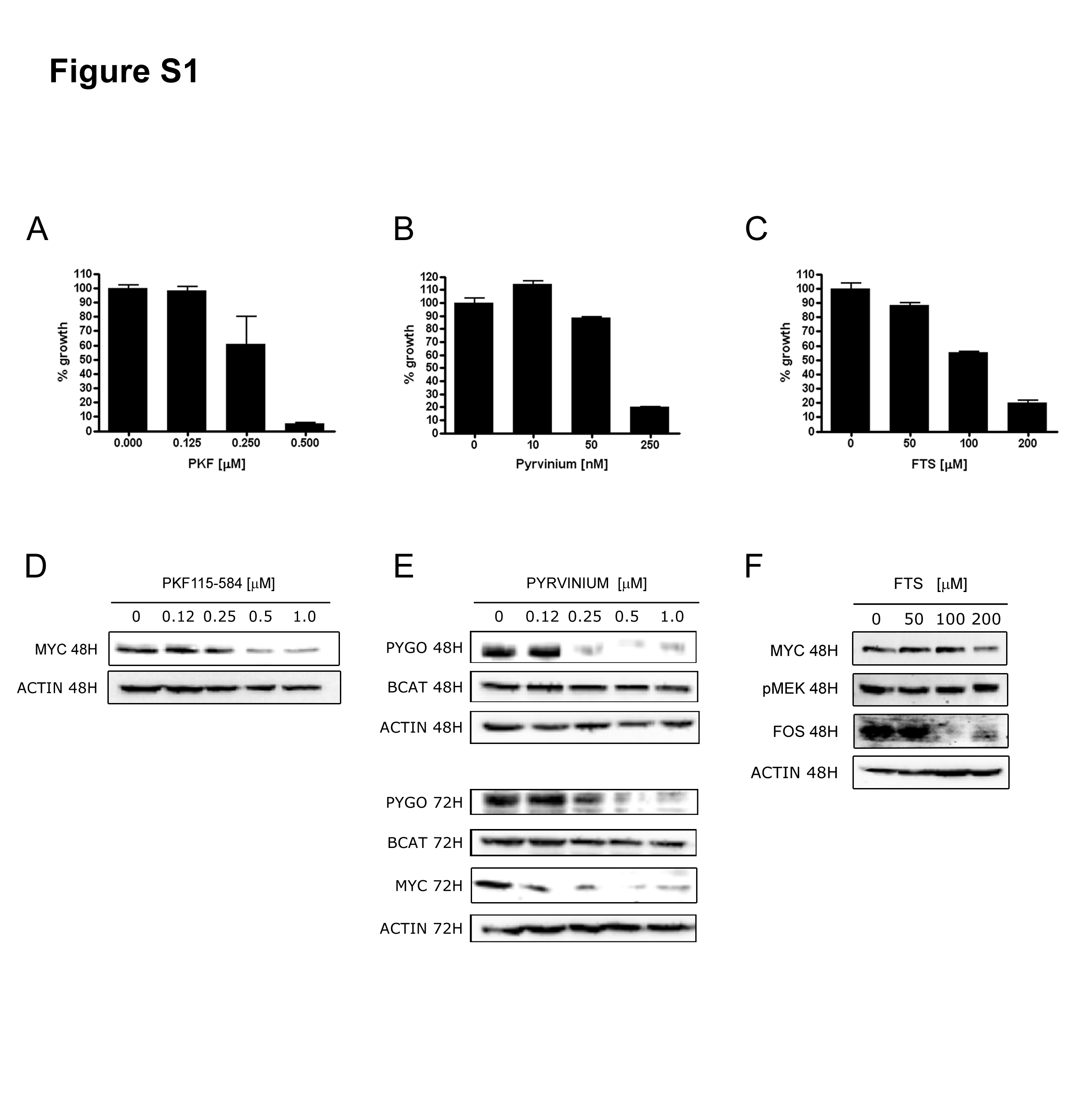

Supplement: Figure S1 — Effects of PKF115-584, Pyrvinium and FTS treatments in DLD-1 cells. The cells were treated with increasing concentrations of PKF115-584 (A, D), pyrvinium (B, E) or FTS (C, F). Cell growth was measured at 72 hours by MTS assay (A–C). Total lysates were probed with the indicated antibodies (D–F). (TIF) [file pone.0051449.s001.tif]

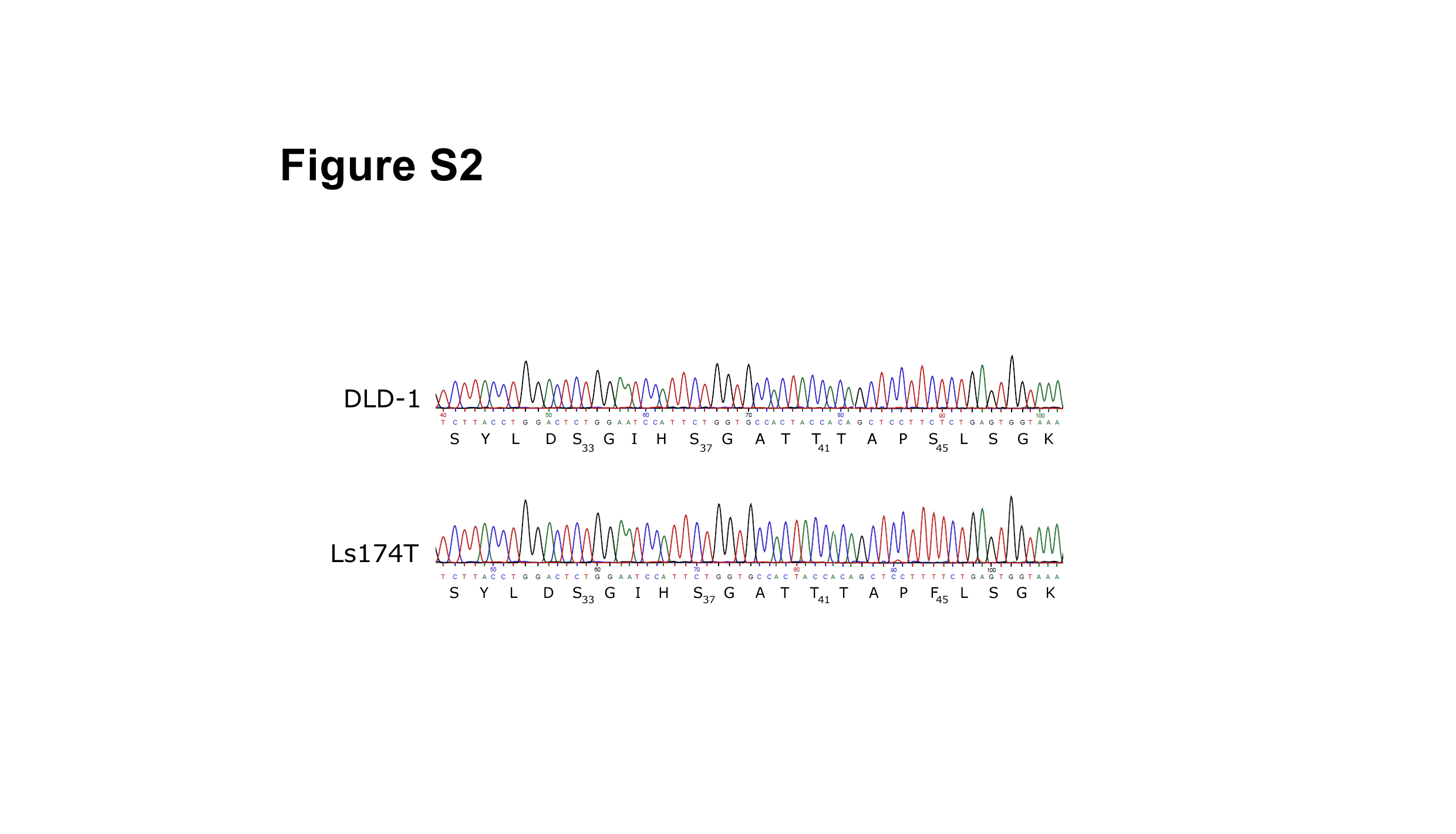

Supplement: Figure S2 — Sequence of the N-terminal b-catenin region in DLD-1 and Ls174T cells. The known CK1α and GSK3β target aminoacids 33, 37, 41 and 45 are indicated. Mutation of Ser45 to Phe is confirmed in Ls174T cells. (TIF) [file pone.0051449.s002.tif]

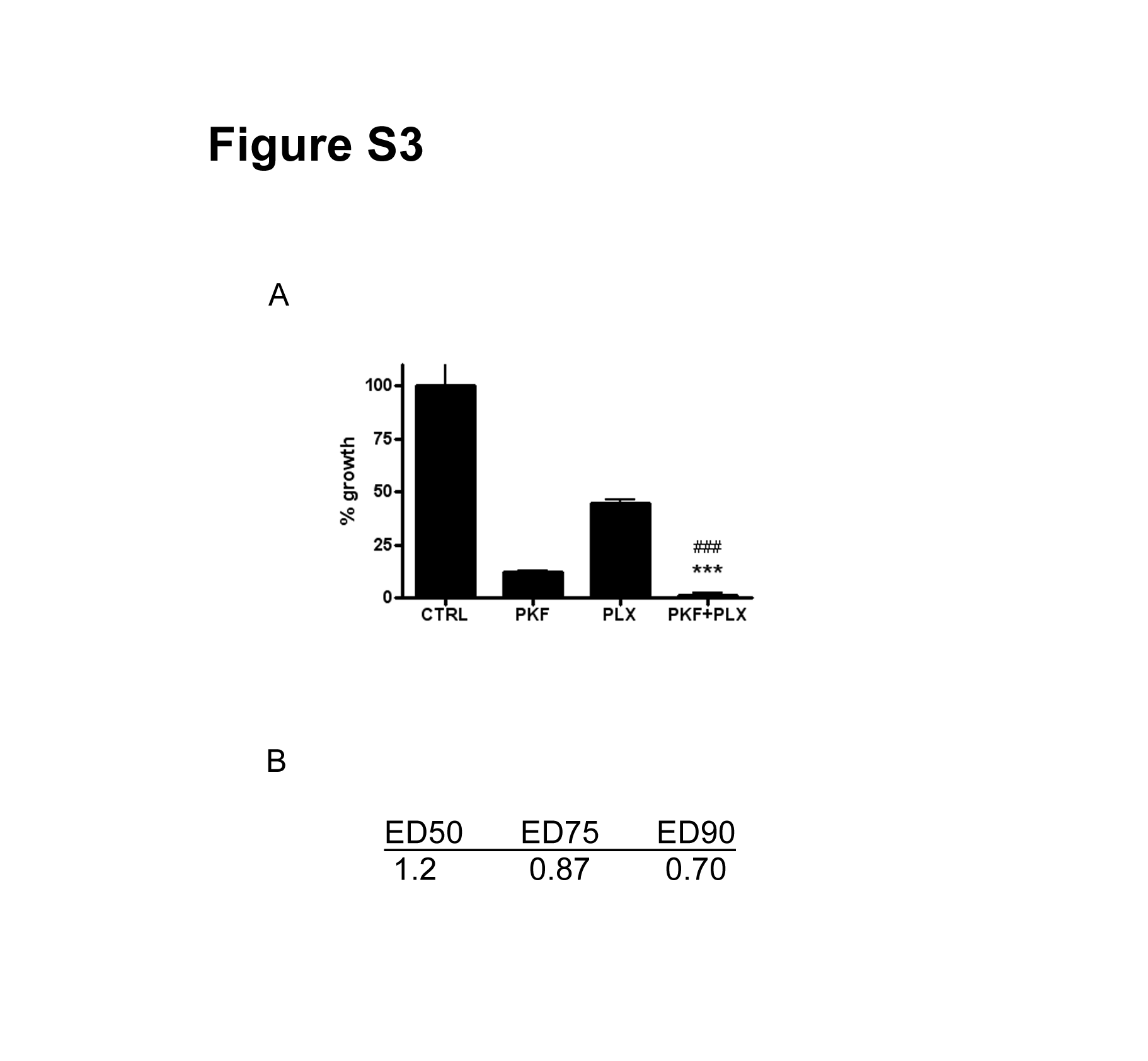

Supplement: Figure S3 — Effects of PKF115-584/PLX-4032 combination in HT-29 cells. (A) The cells were cultured for 6 days in the presence of inhibitors as single agents or in combination (PKF = 0.5 μM; PLX = 2 μM). Percent growth was evaluated by MTS assay. (B) Combination Index values were calculated by CalcuSyn. (TIF) [file pone.0051449.s003.tif]

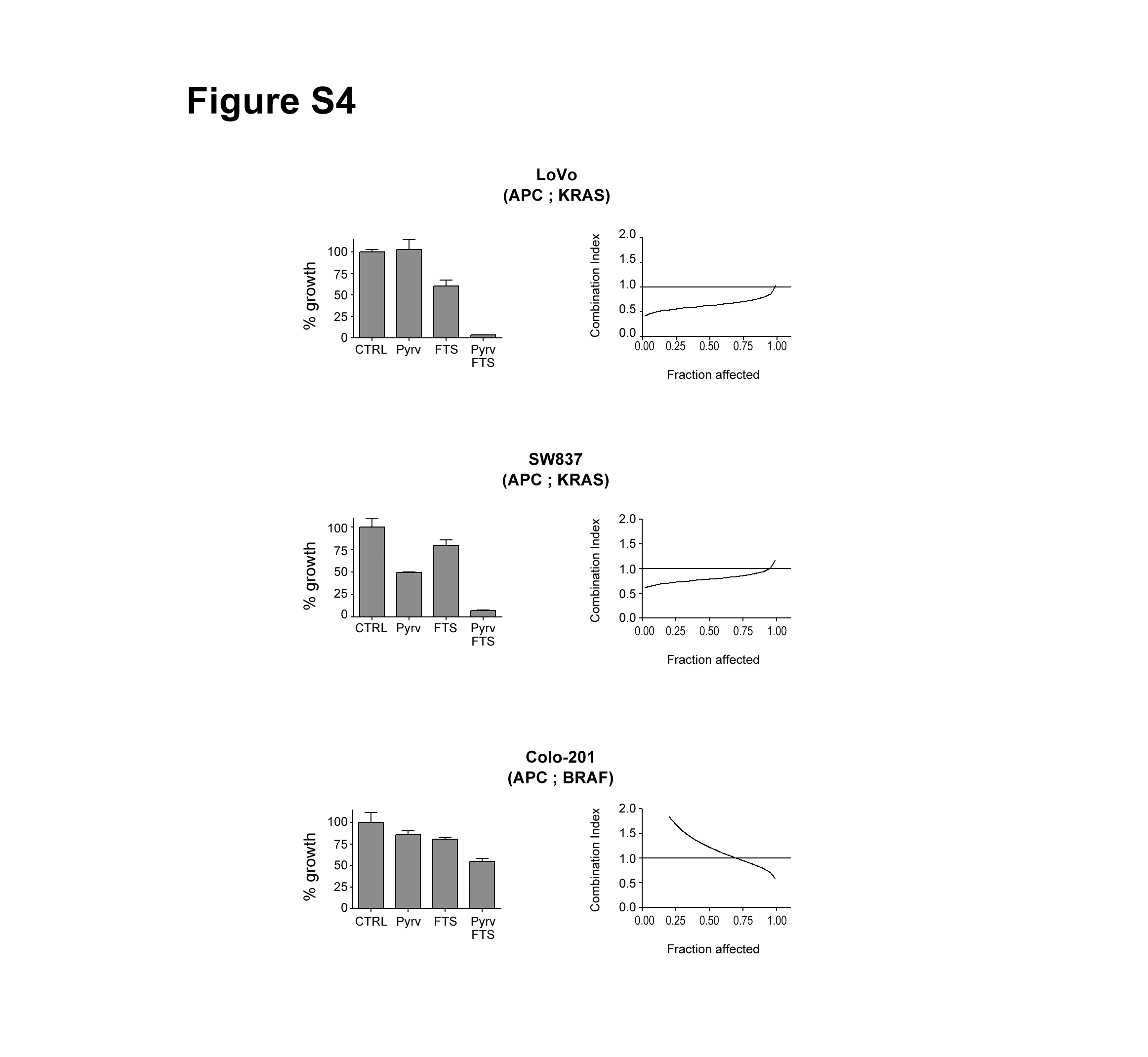

Supplement: Figure S4 — Synergistic effects of pyrvinium/FTS combination in LoVo, SW837 and Colo-201 cell lines. The cells were cultured for 6 days in the presence of inhibitors and then assayed by MTS as described in figure 2. Fractional effect graphs confirm synergism in KRAS-mutated cell lines LoVo and SW837 but not in BRAF-mutated Colo-201. (TIF) [file pone.0051449.s004.tif]

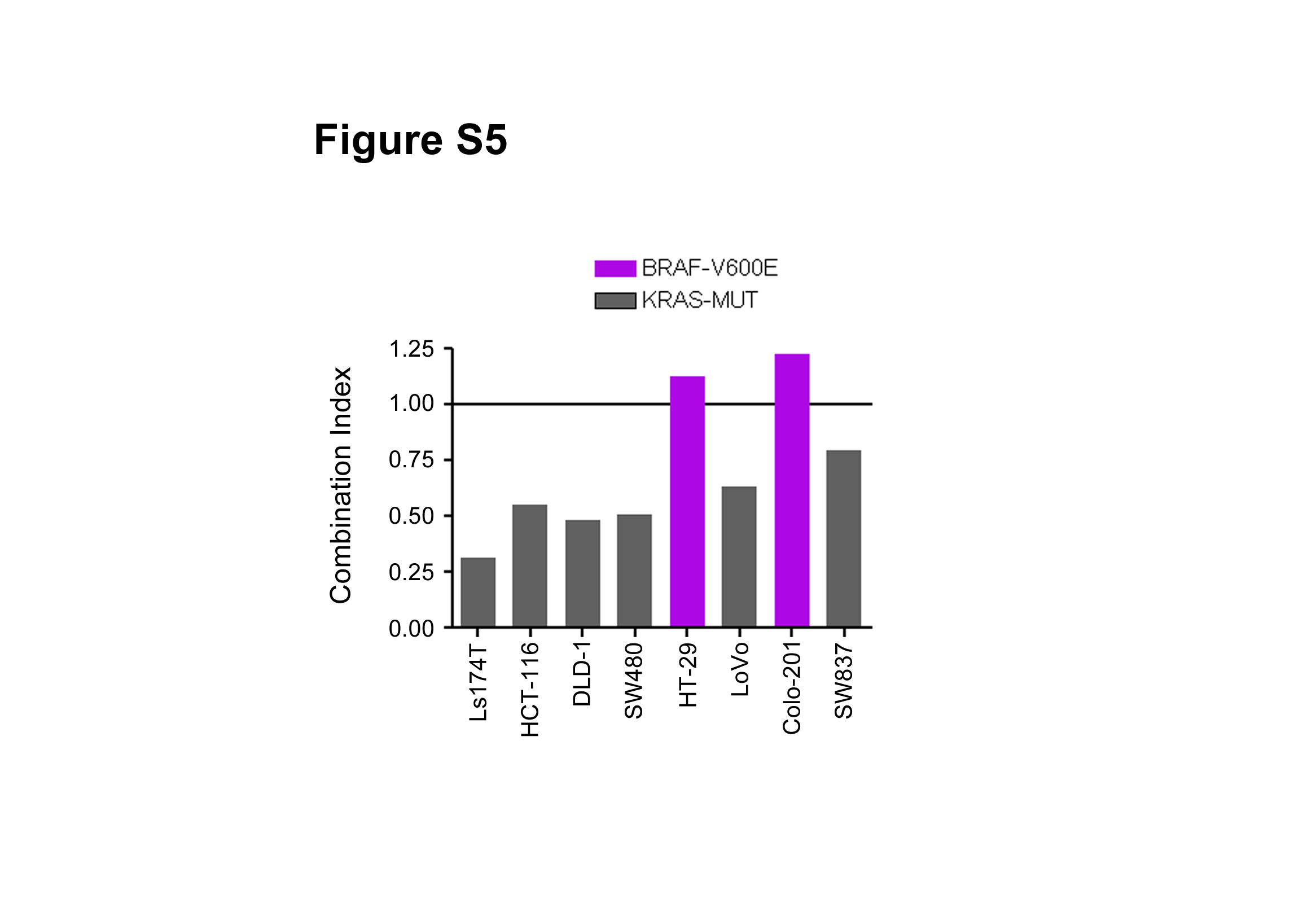

Supplement: Figure S5 — Summary of Combination Index values at ED50 for the pyrvinium/FTS combination. BRAF-mutated cell lines are shown in purple. (TIF) [file pone.0051449.s005.tif]

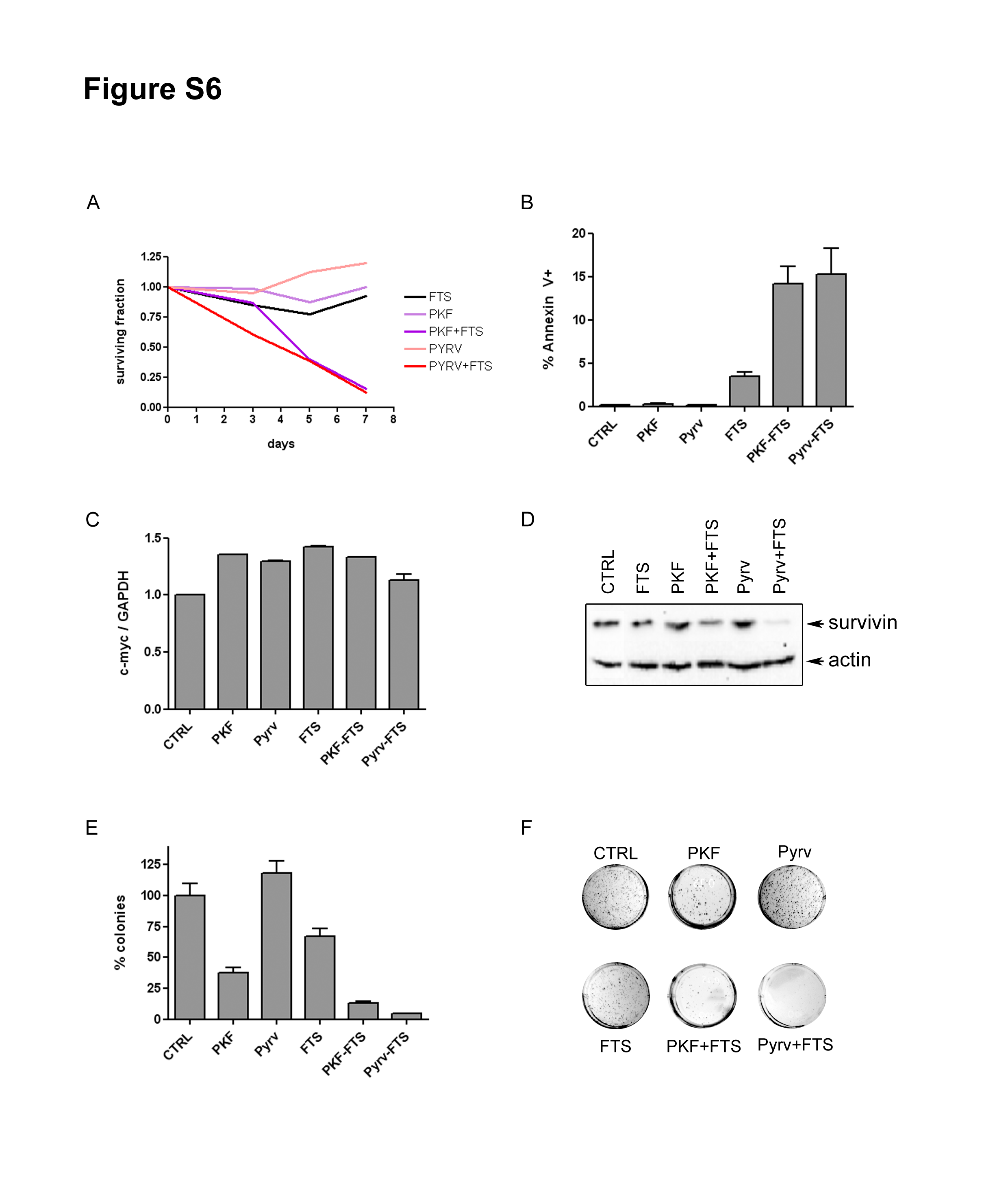

Supplement: Figure S6 — Characterization of synergism in DLD-1 cells. (A) Cells were treated with 100 μM FTS, 125 nM PKF115-584 or 50 nM pyrvinium, alone or in combinations, or vehicle, for 7 days. Cell culture viability relative to control was assessed by MTS. (B) Apoptosis was determined by annexin V/propidium iodide double staining after 72 hours treatment as indicated. (C) c-myc expression evaluated by real-time PCR after 24 hours incubation with the indicated compounds, using GAPDH as a reference gene. Expression was normalized on vehicle-treated control cells. (D) The cells were incubated for 72 hours with inhibitors and cell lysates probed with anti-survivin and β-actin antibodies. (E–F) Ten thousand cells were grown in soft-agar in the presence of PKF115-584 (0.25 μM), pyrvinium (25 nM), FTS (100 μM), alone or combined. Large colonies were counted 20 days later. The number of colonies formed by untreated control cells is set as 100% (E). Representative photographs are shown in panel F. (TIF) [file pone.0051449.s006.tif]

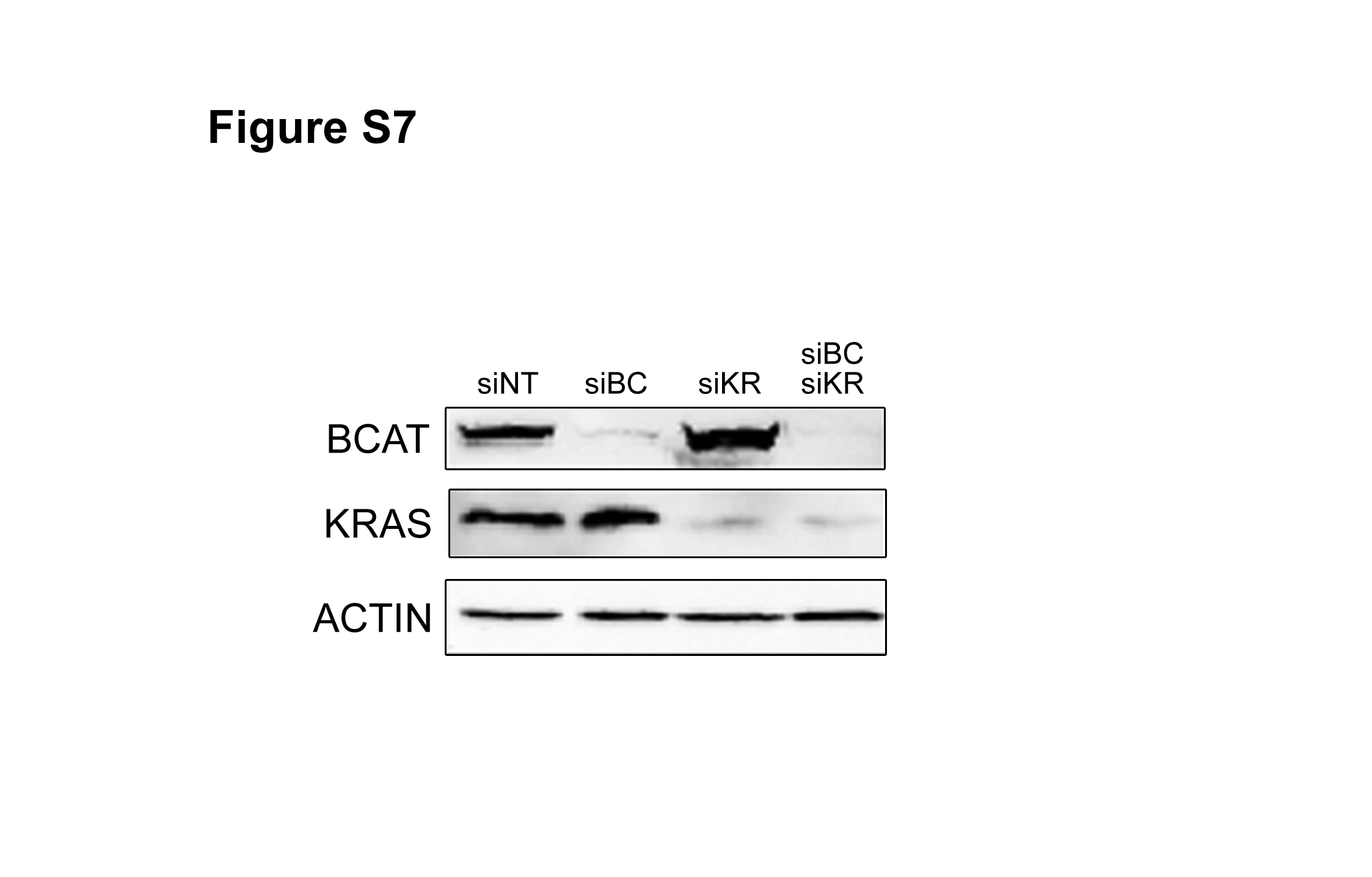

Supplement: Figure S7 — Western blot analysis of siRNA target genes. Ls174T cells stably expressing dox-inducible shRNAs were treated for 3 days with doxycycline. Total cell lysates were probed with anti-β-catenin (BCAT), anti-KRAS or anti-actin antibodies. siNT, non-targeting siRNA; siBC, anti-β-catenin siRNA; siKR, anti-KRAS siRNA. (TIF) [file pone.0051449.s007.tif]

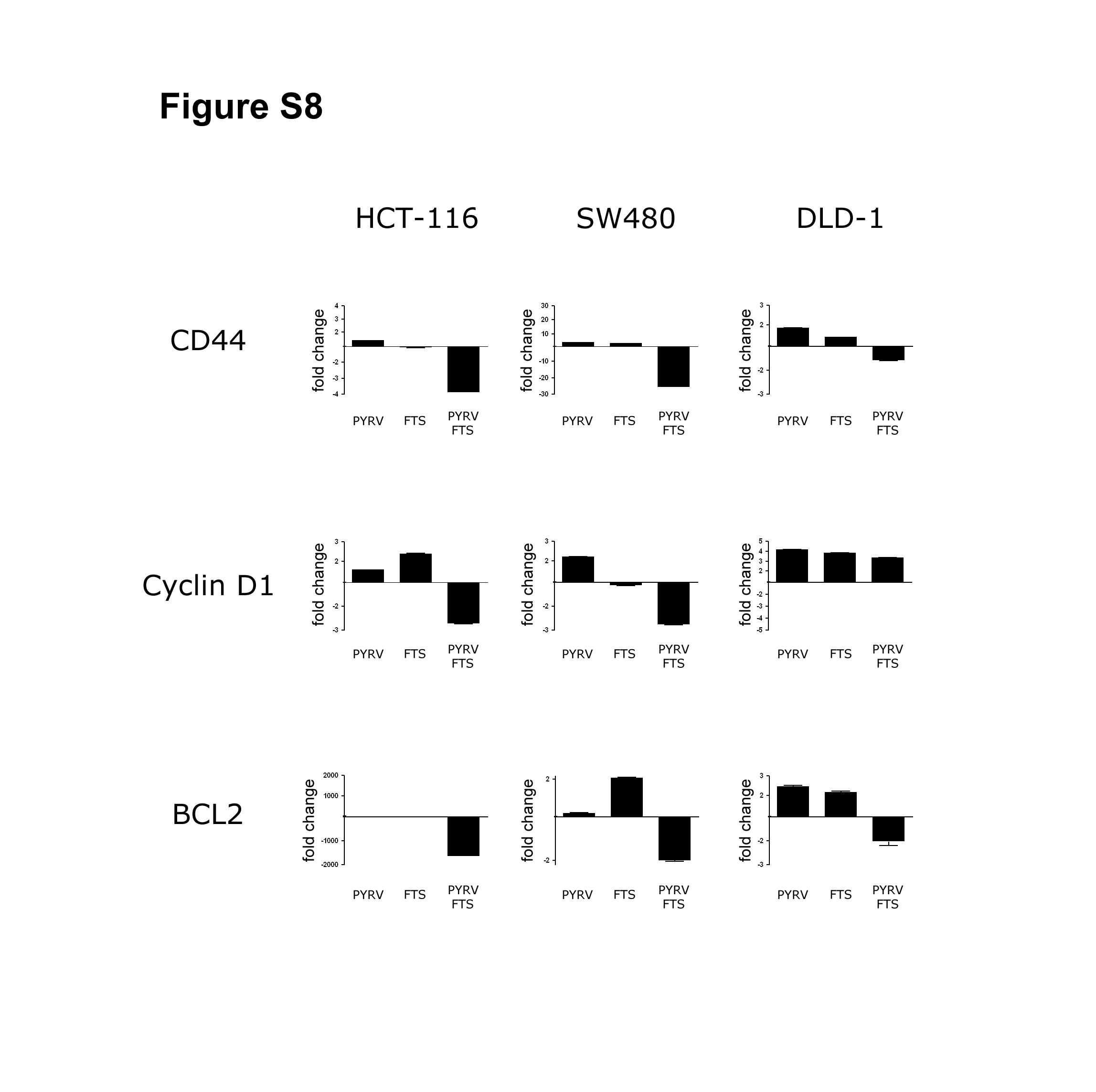

Supplement: Figure S8 — CD44, Cyclin D1 and BCL2 expression fold change obtained in DLD-1, SW480 and HCT-116 cells. The cells were treated for 72 hours with the indicated compounds. Gene expression was analyzed by real-time PCR as in figure 4B. PYRV = pyrvinium. (TIF) [file pone.0051449.s008.tif]

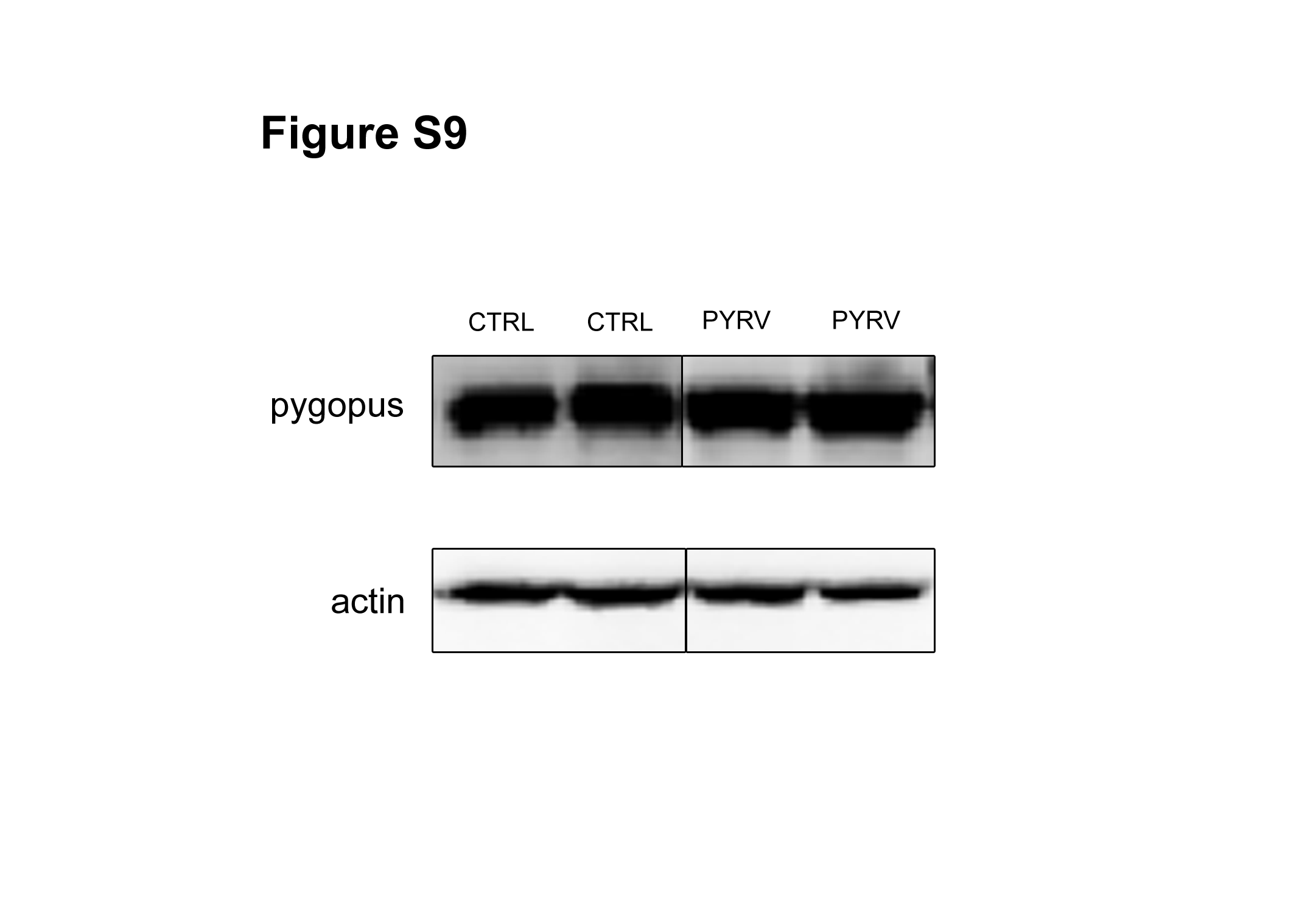

Supplement: Figure S9 — Repeated oral administration of pyrvinium pamoate (10 mg/kg q.d. for 3 days) did not cause pygopus down-modulation in nude mice. Two control mice and two treated mice are shown. A marker lane running in the middle of the gel has been deleted. Actin is shown as a loading control. (TIF) [file pone.0051449.s009.tif]
