# Supplementary material for: Synergistic Effects of Combined Wnt/KRAS Inhibition in Colorectal Cancer Cells
Source: PLoS One. 2012 Dec 5;7(12):e51449. doi: 10.1371/journal.pone.0051449 (PMC3515485; doi:10.1371/journal.pone.0051449)
Supplement: Table S1 — Mutations in the genes encoding APC, β-catenin, KRAS, BRAF and p53, and microsatellite instability status (MSI) of CRC cell lines used in this study. (DOC) [file pone.0051449.s010.doc]

**Table S1.** Mutations in the genes encoding APC, β-catenin, KRAS, BRAF and p53, and microsatellite instability status (MSI) of CRC cell lines used in this study*.

| **Cell Line** | **APC** | **CTNNB1** | **KRAS** | **BRAF** | **TP53** | **MSI** |
| --- | --- | --- | --- | --- | --- | --- |
| **Colo-201** | E1554fs | N287S | wt | V600E | wt | - |
| **DLD-1** | G1416fs | wt | G13D | wt | S241F | + |
| **HCT-116** | wt | S45del | G13D | wt | wt | + |
| **HT-29** | E1154fs/E853* | wt | wt | V600E | R273H | - |
| **LoVo** | T1430fs/R1114* | wt | G13D | wt | wt | + |
| **Ls174T** | wt | S45F | G12D | wt | wt | + |
| **SW480** | Q1338* | wt | G12V | wt | R273H/P309S | - |
| **SW837** | R1450*/R213* | wt | G12C | wt | R248W | - |

*Data source:

CCLE (<http://www.broadinstitute.org/ccle/home>)

COSMIC (<http://cancer.sanger.ac.uk/cancergenome/projects/cosmic/>)

IARC (<http://www.iarc.fr/>)

Umar A, et al., (1994) J Biol Chem 269: 14367-14370

Gayet J, et al., (2001) Oncogene 20: 5025-5032

Ilyas M, et al. (1997) Proc Natl Acad Sci U S A 94: 10330-10334

Casares S, et al. (1995) Oncogene 11:2303-2310

Tsushimia T, et al. (2001) Cancer Genet Cytogenet 126: 34–38

Smakman N, et al. (2006) Cancer Res 10: 5403-5408
