# Supplementary material for: Synergistic Effects of Combined Wnt/KRAS Inhibition in Colorectal Cancer Cells
Source: PLoS One. 2012 Dec 5;7(12):e51449. doi: 10.1371/journal.pone.0051449 (PMC3515485; doi:10.1371/journal.pone.0051449)
Supplement: Table S2 — Primers used for QPCR analysis. The common protein name is provided in brackets along with the official gene symbol, when different. (DOC) [file pone.0051449.s011.doc]

**Table S2.** Primers used for QPCR analysis. The common protein name is provided in brackets along with the official gene symbol, when different.

| **GENE ID**  **(alias)** | **FORWARD** | **REVERSE** |
| --- | --- | --- |
| **APAF1** | GCCAAGCAGGAGGTCGATAATG | GACCATCCTCAGAAAAGCAGGC |
| **AXIN2** | CAAACTTTCGCCAACCGTGGTTG | GGTGCAAAGACATAGCCAGAACC |
| **BAK1** | TTACCGCCATCAGCAGGAACAG | GGAACTCTGAGTCATAGCGTCG |
| **BAX** | ATGATTGCCGCCGTGGACA | CAACCACCCTGGTCTTGGATC |
| **BCL2** | TGCACCTGACGCCCTTCAC | AGACAGCCAGGAGAAATCAAACAG |
| **BCL9L** | GACAGTGAGGAGGACGACAAGCC | CCTGGCACGCTGCTCTCGCT |
| **BCL2L1**  **(Bcl_XL_)** | GTAAACTGGGGTCGCATTGT | TGCTGCATTGTTCCCATAGA |
| **BIK** | GGAGGTTCTTGGCATGACTGAC | TGAGGCTCACGTCCATCTCGTC |
| **CD44** | CCAGAAGGAACAGTGGTTTGGC | ACTGTCCTCTGGGCTTGGTGTT |
| **CDH1**  **(E-cadherin)** | GCCTCCTGAAAAGAGAGTGGAAG | TGGCAGTGTCTCTCCAAATCCG |
| **COX2** | CGGTGAAACTCTGGCTAGACAG | GCAAACCGTAGATGCTCAGGGA |
| **CTBP2** | TTTTGTGGGCTGGTTGGGAGAG | CAGGCATCGTGGGTGTGG |
| **CCND1**  **(Cyclin D1)** | GCTGGAGCCCGTGAAAAAGA | CTCCGCCTCTGGCATTTTG |
| **CCND2**  **(Cyclin D2)** | GAGAAGCTGTCTCTGATCCGCA | CTTCCAGTTGCGATCATCGACG |
| **TNFRSF25**  **(DDR3)** | CAACTCCACCTGCCTTGTGTGT | CCACTGCTGAACAGTTCTCCAG |
| **FAS** | GGACCCAGAATACCAAGTGCAG | GTTGCTGGTGAGTGTGCATTCC |
| **FOS** | GCCTCTCTTACTACCACTCACC | AGATGGCAGTGACCGTGGGAAT |
| **FOSL1**  **(Fra1)** | GGAGGAAGGAACTGACCGACTT | CTCTAGGCGCTCCTTCTGCTTC |
| **GAST**  **(Gastrin)** | CCTCTCATCATCGAAGGCAGCT | CTTCTTCCTCCAGCCATGGTCC |
| **TCF4**  **(ITF-2)** | CAACGGGACAGACAGTATAATGG | ATGGAGGAGAGCCAACAGGAGT |
| **JUN** | CCTTGAAAGCTCAGAACTCGGAG | TGCTGCGTTAGCATGAGTTGGC |
| **KRAS** | CAGTAGACACAAAACAGGCTCAG | TGTCGGATCTCCCTCACCAATG |
| **ALOX15** | ACCTTCCTGCTCGCCTAGTGTT | GGCTACAGAGAATGACGTTGGC |
| **MCL1** | CCAAGAAAGCTGCATCGAACCAT | CAGCACATTCCTGATGCCACCT |
| **PMAIP1**  **(Noxa)** | TCCAGCAGAGCTGGAAGTCGAGTGT | ATGAATGCACCTTCACATTCCTCT |
| **CDKN1A**  **(p21)** | AGGTGGACCTGGAGACTCTCAG | TCCTCTTGGAGAAGATCAGCCG |
| **CDKN1B**  **(p27)** | ATAAGGAAGCGACCTGCAACCG | TTCTTGGGCGTCTGCTCCACAG |
| **RPS6KB1**  **(p70S6K1)** | TATTGGCAGCCCACGAACACCT | GTCACATCCATCTGCTCTATGCC |
| **RPS6KB2**  **(p70S6K2)** | ACACCTTCTGCGGCACCATTGA | CGATCCAGTGAGCATGTCGTAC |
| **PPARG** | AGCCTGCGAAAGCCTTTTGGTG | GGCTTCACATTCAGCAAACCTGG |
| **PKCA** | GCCTATGGCGTCCTGTTGTATG | GAAACAGCCTCCTTGGACAAGG |
| **RASSF7** | ACCTGCCAGGAAGTGGTCATCG | ACTGGACACTCTTGTGGCAGCA |
| **SP1** | ACGCTTCACACGTTCGGATGAG | TGACAGGTGGTCACTCCTCATG |
| **SRC** | CTGCTTTGGCGAGGTGTGGATG | CCACAGCATACAACTGCACCAG |
| **TCF7L2**  **(Tcf-4)** | CATGCCGCAGCTGAACGGCGGT | TCATTCCGCCTCGGAATCGGAGGAG |
| **WISP** | GCAGGGAAGAAGTGTCTGGCTGTG | GGGTTGATAGGAGCGTGTGCTGA |
| **XIAP** | TGGCAGATTATGAAGCACGGATC | AGTTAGCCCTCCTCCACAGTGA |
| **MYC** | CTGTATGTGGACGGCTTCTCG | CTGCTGTCGTTGAGAGGGTAG |
